# Determination of an Initial Stage of the Bone Tissue Ingrowth Into Titanium Matrix by Cell Adhesion Model

**DOI:** 10.3389/fbioe.2021.736063

**Published:** 2021-09-13

**Authors:** Ziyu Liu, Maryam Tamaddon, Shen-Mao Chen, Haoyu Wang, Vee San Cheong, Fangli Gang, Xiaodan Sun, Chaozong Liu

**Affiliations:** ^1^ Division of Surgery & Interventional Science, University College London, Royal National Orthopaedic Hospital, London, United Kingdom; ^2^ School of Engineering Medicine, Beihang University, Beijing, China; ^3^ Key Laboratory of Advanced Materials of Ministry of Education of China, School of Materials Science and Engineering, Tsinghua University, Beijing, China; ^4^ Insigno Institute of in Silico Medicine and Department of Automatic Control and Systems Engineering, University of Sheffield, Sheffield, United Kingdom

**Keywords:** osteochondral scaffold, gradient design, volume of fluid model, discrete phase model, cell adhesion

## Abstract

For achieving early intervention treatment to help patients delay or avoid joint replacement surgery, a personalized scaffold should be designed coupling the effects of mechanical, fluid mechanical, chemical, and biological factors on tissue regeneration, which results in time- and cost-consuming trial-and-error analyses to investigate the *in vivo* test and related experimental tests. To optimize the fluid mechanical and material properties to predict osteogenesis and cartilage regeneration for the *in vivo* and clinical trial, a simulation approach is developed for scaffold design, which is composed of a volume of a fluid model for simulating the bone marrow filling process of the bone marrow and air, as well as a discrete phase model and a cell impingement model for tracking cell movement during bone marrow fillings. The bone marrow is treated as a non-Newtonian fluid, rather than a Newtonian fluid, because of its viscoelastic property. The simulation results indicated that the biofunctional bionic scaffold with a dense layer to prevent the bone marrow flow to the cartilage layer and synovia to flow into the trabecular bone area guarantee good osteogenesis and cartilage regeneration, which leads to high-accuracy *in vivo* tests in sheep . This approach not only predicts the final bioperformance of the scaffold but also could optimize the scaffold structure and materials by their biochemical, biological, and biomechanical properties.

## Introduction

Knee osteoarthritis is a common and frequently occurring disease in the middle-aged and elderly population, including cartilage destruction and subchondral bone thickening (Peat et al., 2001). It ranks first in causing disability in the elderly and seriously affects the quality of life. Large-area cartilage defects can only be treated by joint replacement surgery. In the tissue engineering field, scaffolds play a pivotal role in tissue engineering, which provide a three-dimensional template for cell seeding, temporary mechanical function, and an extracellular matrix environment for tissue regeneration (Karageorgiou and Kaplan, 2005; Hollister, 2006). Osteochondral scaffolds act as an osteoconductive part and serve as delivery vehicles for cytokines like bone morphogenetic proteins (BMPs), thus providing osteoinduction (Groeneveld et al., 1999). Osteogenesis and bone mesenchymal stem cell (BMSC) attachment occur after the implantation of the scaffolds.

**GRAPHICAL ABSTRACT:**
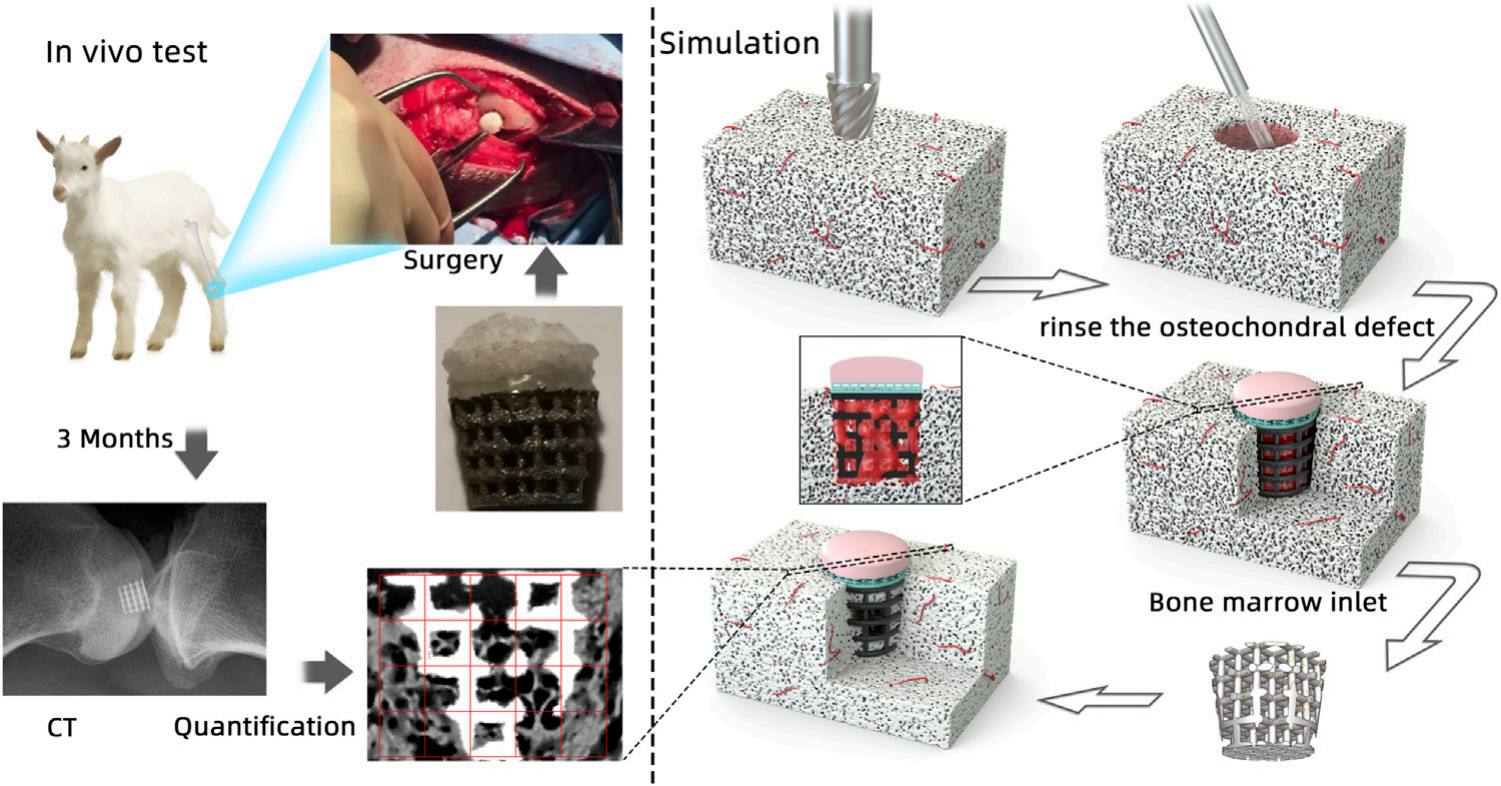


Aiming for osteogenesis and cartilage regeneration, an ideal scaffold is needed to provide suitable biomimetic mechanical and biological environments having similar morphology and function like natural osteochondral bone which could optimize integration into neighboring tissues (Healy, 1999; Hubbell, 1999; Sakiyama-Elbert and Hubbell, 2001; Lin et al., 2004; Karageorgiou and Kaplan, 2005; Wu et al., 2017). In general, *in vitro* and *in vivo* tests are used to evaluate scaffold bioperformance with high funding cost. An ethical and economical method is needed to evaluate scaffold bioperformance which considers the influence of the material (surface tension and material kinds) and its geometry (pore size, porosity, geometry, and surface area).

Computational analysis is seen as a promising method for scaffold evaluation. Sanz-Herrera et al. analyzed bone growth and formation in a two-dimensional (2D) scaffold by bone remodeling theories which assumed that it is driven by a mechanical stimulus (Beaupré et al., 1990; Adachi et al., 2001; Sanz-Herrera et al., 2008). With the same assumption, Adachi et al. also used the bone remodeling theory with uniform stress hypothesis to express how new bone tissues formed (Cowin, 1993; Adachi et al., 1998; Adachi et al., 2006). However, neither of them involved fluid stimuli in the simulation, which plays an important role in bone tissue regeneration when using an osteochondral scaffold. Moreover, recruiting BMSCs, as the first step of tissue engineering, and simulating BMSC attachment on the scaffold and their distribution is a really significant step in predicting the final bioperformance (Byrne et al., 2007).

Prendergast et al. proposed a theory that mechanical and fluid mechanical stimuli caused stem cell differentiation (Prendergast et al., 1996). And this mechano-regulation method successfully validated tissue differentiation by experimentally surrounding implants (Huiskes et al., 1997). Damian P.B. et al. explored various design parameters of the scaffold in tissue regeneration, and by this method modeling, cell proliferation, migration, and differentiation, he assumed that cells were randomly seeded on the lattice scaffold. However, cells distributed on the scaffold are not random, and it is determined by a lot of complex influence factors—material, surface roughness, geometry, and bone marrow flow speed. After recruiting the scaffold, BMSCs would start proliferating and migrating until they are mature enough to undergo differentiation. The position of cells would finally influence the final tissue formation (Wendt et al., 2006; Santoro et al., 2010). Till now, few researchers have studied cell distribution in the scaffold at the initial stage of the tissue engineering process after surgery since it is impossible to sacrifice animals at the initial stage to observe cell distribution ethically and economically. In that case, computational analysis provided an important solution to investigate this postsurgery process. As it is impossible to observe cell distribution using *in vivo* tests to validate simulation results, our previous work (Liu et al., 2020) used a numerical cell attachment model with a volume of fluid (VOF) model and discrete phase model (DPM) to investigate the cell seeding process and validated using *in vitro* tests.

To explore the relationship between BMSC distribution at the postsurgery initial stage and bone formation on the scaffold, the novel model is developed in a more real environment for cell attachment. The bone marrow is set as non-Newtonian fluid calculated by the power law for non-Newtonian viscosity, rather than normal Newtonian fluid like the solution for cell seeding. The model is validated by using *in vivo* tests in which bone formation and distribution are quantified by a self-designed MATLAB program by analyzing the Micro-CT–scanned image by threshold-based operation. The information of osteogenesis could be obtained not only on the outside of the scaffold but also within the pores of the scaffold. This model is aiming to decrease both *in vivo* and *in vitro* tests for optimizing the scaffold design and material properties which could avoid unnecessary time and money investment.

## Materials and Methods

### Bionic Scaffold and Manufacturing

**FIGURE 1:**
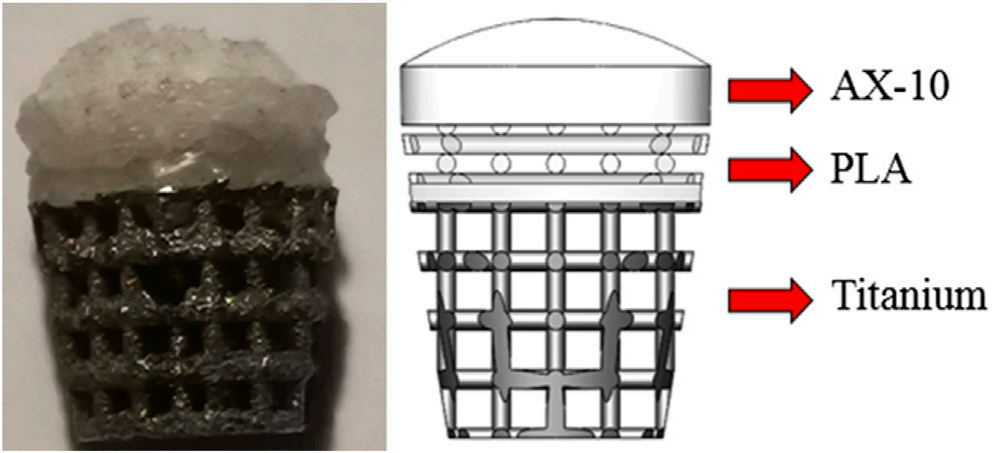
Biomimetic multi-layer gradient osteochondral scaffold (**left:** real structure; **right:** schematic structure).

The bionic scaffold has 3 layers shown in Figure 1. The top layer is produced by 10% poly(lactic-co-glycolic acid) (PLGA) solutions in acetone pipetted into 90% freeze-dried collagen scaffold which is called AX-10. The cone structure is combined with an 8.5-mm-diameter and 1.2-mm-height spherical cap on the top and an 8.5-mm-diameter and 1.5-mm-height column at the bottom. The medium layer is 3D-printed with polylactic acid (PLA) composited by one solid dense layer at the bottom and one high porosity truncated cone structure. The high-porosity structure has 4 small layers, and each layer’s column beam has the same direction but perpendicular to the close layer’s beam direction. The column size is 0.5 mm diameter. The PLA scaffold was plasma-treated at 50–60 Hz frequency and with 60% power-oxygen for 3 min on two sides (top and bottom). The samples were then sterilized with 70% ethanol for 15 min. The bottom layer with a high-porosity and high interconnected pore network was printed by an EOS M270 machine with titanium powder. The high-porosity and interconnected structure aims to provide enough routes for nutrient transport like porous trabecular bones.

For large osteochondral defect repair, the PLGA layer is a column design with 8.5 diameter and 1.5 mm height. Below the porous structure, the dense PLA layer is designed as a 0.5-mm-height column with 8 mm diameter. The Ti layer is designed as a truncated cone with 8 mm diameter on the top surface and 5.9 mm on the bottom surface manufactured by an EOS 290 3D printer. To combine three layers together, the PLA layer was melted and pressed into a titanium matrix and fused together by hot fusion. And then, these two layers were submerged into the cross-linked collagen suspension and then freeze-dried.

### *In Vivo* Test

#### Ethical Aspects and Animals

Five young female sheep with a mean weight of 81.6 ± 6.4 kg were operated in the Royal Veterinary College (RVC). All sheep were treated according to Animals (Scientific Procedures) Act (ASPA). Animal examinations, housing, feeding, and veterinary care were conducted using established procedures. The sheep were housed in a free land with sufficient food and water. Furthermore, sheep can freely move in the outside during the research period.

#### Surgical Procedure

Under anesthetics, the sheep were bedded carefully on their right side, exposing their left knee. A truncated critical-sized osteochondral defect of 9 mm diameter was created using two surgical drills on the load-bearing area medial to the femoral condyle up to a depth of 10 mm.

**FIGURE 2:**
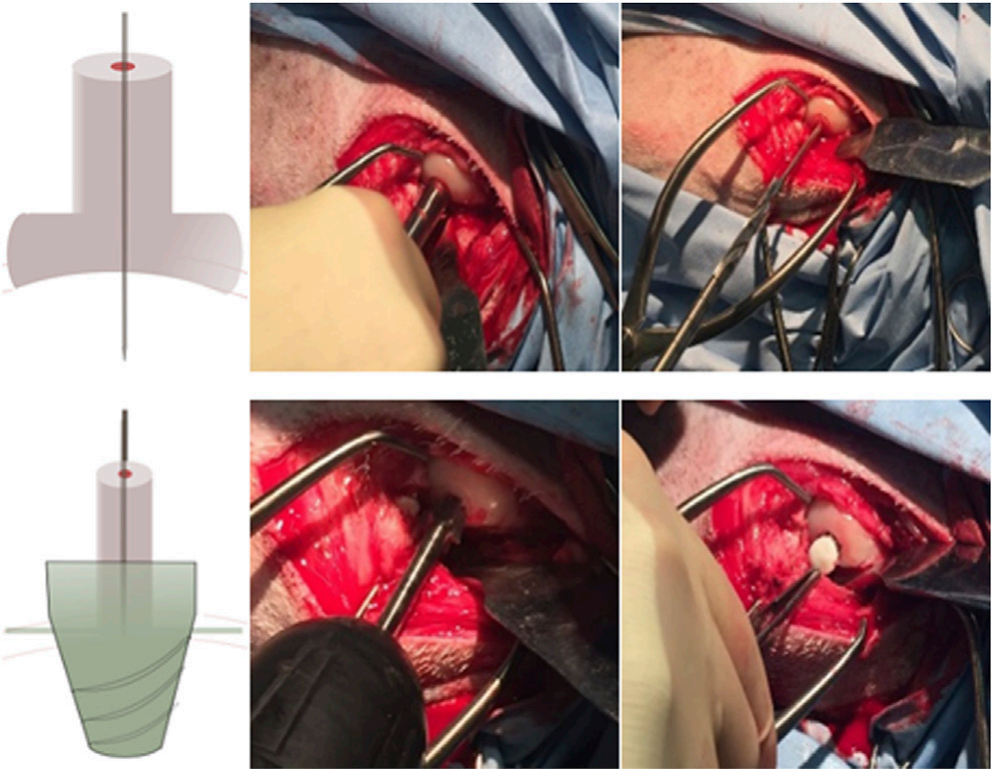
Presurgical operation procedure: 1–3. Drill the small hole located by a drill sleeve and insert a pin guide into the hole; 4–5. Access the driller bite to create a larger hole, and use special design roam to create a truncated cone hole; and 6. Place the mountain scaffold into the osteochondral defect.

According to the surgical operation procedure (Figure 2), first, a small hole was created by using a drill sleeve on the load-bearing area medial to the femoral condyle, and then a nail guide was put into the bone with a 9.3-mm-deep hole. After that, the cartilage of 9.45 mm diameter was removed using a circular cylindrical cutter. Then, a critical-sized, truncated cone-shaped osteochondral defect was drilled up to 8.8 mm depth by using two surgical drills . The truncated cone defect was scoured by water until the biomimic multi-layer gradient scaffold was inserted into the defect.

After the surgery, they were housed in individual places for five days restricting their activities at the initial stage of the healing process. In these five postsurgery days, they were treated with analgesia (carprofen 5 mg/kg) and antibiotics (enrofloxacin 10 mg/kg) subcutaneously twice daily. After 3 months, the animals were euthanized under anesthesia. Both legs’ condyles were fixed with paraformaldehyde at room temperature for further analysis.

#### Micro-Computed Tomography (Micro-CT) and X-Ray Analysis

The X-ray micrographs of limbs were used to look at the stability of scaffolds in the joint. The X-ray scan of pre-euthanized and the postoperative tissue scaffold was performed by using a Nikon XT H 225 machine which offers a powerful 225 kV micro-focus source with real-time X-ray visualization. And the tissue with the scaffold inside was scanned in a small-scale slice-by-slice manner with great resolution. Three-dimensional reconstruction was solved by CTvox and CTan (Bruker, United States) software. The knee joint of the animal was scanned using X-ray immediately after euthanasia to look at the position of the scaffolds within the joint after 3 months.

#### X-Ray–Based Bone Analysis for Quantification of Osteogenesis and Bone–Scaffold Interaction

2D images from X-ray–scanned objects were utilized to determine the amount of the newly formed bone in the scaffold and bone–scaffold surface interaction values. Due to the high resolution of the scanner, the information within the pores of the scaffolds could be obtained with certainty. Thus, not only the outer but also the inner regions of the scaffolds were taken into account for the determination of osteogenesis.

**FIGURE 3:**
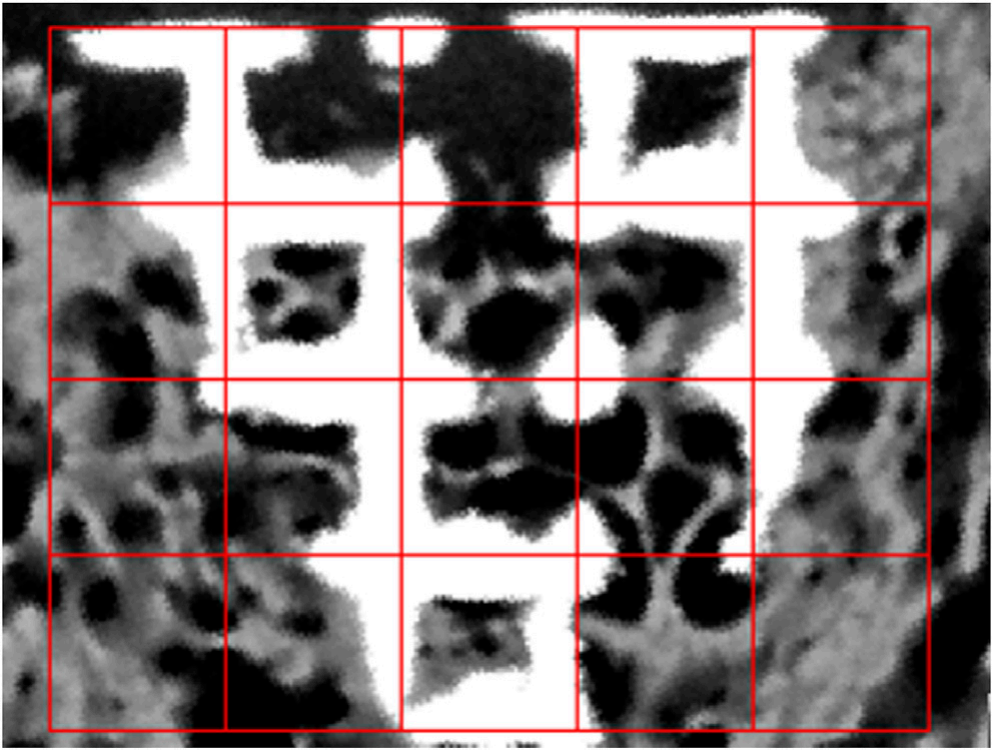
MATLAB program image analysis of osteogenesis.

MATLAB R2017a is used to translate images to binary images at first and then to divide images into the same length grids (Figure 3). As binary images store an unsigned one-byte integer to describe the area between 0 (representing black) and 255 (maximum value—representing white), we defined three threshold values to identify the scaffold and bone structure. As the images are composed of pixels, 10 pixels of the bone and scaffold were selected to define the pixel’s threshold range in the program. To define both ranges accurately, scaffold thresholds are calculated by selecting 10 pixels on the edge of the image (white area) as the pixel in the middle of the scaffold threshold is definitely larger than that in the edge. As for bone threshold definition, 5 pixels with dim color that represent the bone tissue were selected to find the lowest value, and 5 pixels with the lightest color were selected to find the highest value. After careful selection, the thresholds of the scaffold which were seen as white are all beyond 250. And the threshold of the bone could be set between 60 and 180. The thresholding operation is defined as follows:

$$\mathrm{Area}\;\mathrm{defination}\;=\;\{\begin{array}{cc} \mathrm{Titanium}\;\mathrm{scaffold} & \mathrm{,intensity>}\mathrm{250}\\ \mathrm{Bone} & \mathrm{,60}\leq \mathrm{intensity<}\mathrm{180} \end{array}$$

The pixel is seen as bone if the intensity is larger than 60 and smaller than 180. As for the scaffold area percentage, the intensity should be greater than 250. The percentage is defined as follows:

$$P_{b}\;=\;\frac{S_{bone}}{S_{all}-S_{s}}$$

where P_b_ represents the bone percentage of each grid, S_all_ is the whole image area of each grid, and S_bone_ and S_s_ are defined as the bone and scaffold area occupied in each grid, respectively.

**FIGURE 4:**
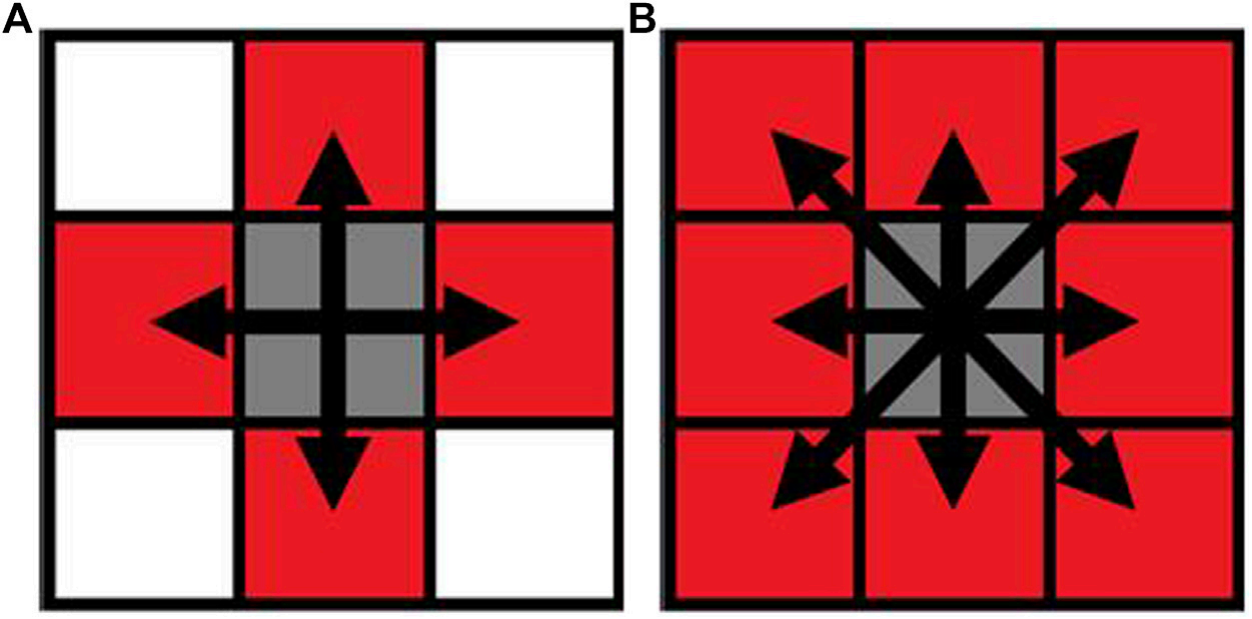
Pixel neighborhood analysis for bone-scaffold connection percentage (**(A)** 4-connected neighborhood; **(B)** 8-connected neighborhood). Simulation of scaffold recruitment for bone marrow mesenchymal stem cells.

There are two different conventions that are used to decide whether pixels (titanium scaffold and bone tissue) are connected or not in two-dimensional images—4-connected and 8-connected neighborhoods. As for the 4-connected neighborhood, pixels are seen as connected when their edges touch. In other words, the pixels, which are along the diagonals, are not considered connected. As for the 8-connected neighborhood, adjoining pixels are connected along not only horizontal and vertical directions but also the diagonal direction. Both are shown in Figure 4.

#### Physical Model and Computational Framework

A column model with a truncated cone hole in the bulk was developed to simulate the scaffold as the same geometry as the osteochondral defect. The osteochondral defect was set as 8.2 mm diameter on the top and 5.88 mm diameter at the bottom which could just fit the bottom of the scaffold but little bit larger than the scaffold's top surface size (8 mm) because this 0.1-mm hole is used for air outlet after the bone marrow flowed into the void space of the defect. To further investigate the relationship between osteogenesis in the scaffold and simulation results of cell distribution, only the titanium scaffold layer is set as a physical model for recruiting BMSCs. This is because only in the titanium layer osteogenesis occurs.

The bone marrow is considered as a continuity fluid, which could carry BMSCs to attach on the scaffold. The volume of fluid (VOF) model is used to control the movements of these two immiscible fluids. Considering BMSCs as discrete particles, cell attachment is governed by the discrete phase model (DPM) with the Stanton–Rutland model through the Eularian–Lagrangian approach. The interaction of the cell with the scaffold is simulated by the cell impingement model (CIM), which is governed not only by cell physical properties (viscosity, surface tension, and density) but also by the impingement conditions (cells velocity and diameter).

**In the DPM,** discrete particles representing the BMSCs were carried by the fluid phase, and trajectories of cells were predicted by integrating the force balance on the cell written in a Lagrangian reference frame. All particles (BMSCs) are set as non-rotating. Particle impingement causes energy loss because of the inelastic collision.

**In the CIM**, the cell impingement model for simulating cell adhesion on the scaffold is defined as three regimes, including stick, rebound, and spread, when cells impinge the scaffold wall. The detailed descriptions are in our previous article.

**In VOF model**, the bone marrow is seen as a non-Newtonian fluid, which was chosen to simulate the fluid of the bone marrow. Air and the bone marrow are governed by the continuity equation and Navier–Stokes equations as follows:

$$\nabla \cdot \vec{u}\;=\;0$$

$$\rho \left(\frac{\partial u}{\partial t}+u\cdot \nabla u\right)\;=\;-\nabla \mathrm{p+p}\vec{g}+\mu \nabla^{2}u$$

Non-Newtonian fluids will be calculated by the power law for non-Newtonian viscosity as follows:

$$\eta \;=\;k\;{\overset{\cdot }{\gamma }}^{\mathrm{n-}1}\;H\left(T\right)$$

where $\overset{\cdot }{\gamma }\;$ is defined as the shear stress rate-of-deformation tensor $\bar{\bar{D}}$:

$$\overset{\cdot }{\gamma }\;=\;\sqrt{\frac{1}{2}\bar{\bar{D}}:\bar{\bar{D}}}$$

$$\bar{\bar{D}}\;=\;\left(\frac{\partial u_{j}}{\partial x_{i}}+\frac{\partial u_{i}}{\partial x_{j}}\right)$$

H(T), known as the Arrhenius law (Wendt et al., 2006; Santoro et al., 2010), is temperature-dependent. As the bone marrow is non-isothermal, H(T) is set to 1.

$$H\left(T\right)\;=\;\exp\left[\alpha \left(\frac{1}{\mathrm{T-}T_{0}}-\frac{1}{T_{\alpha }-T_{0}}\right)\right]$$

BMSCs were treated as spherical particles with non-rotating movement during the calculation and carried by fluid phase (bone marrow). Trajectories of particles were predicted by intergrating force balance on the particles through the Lagrangian frame.

#### Solving Process and Boundary Conditions

**FIGURE 5:**
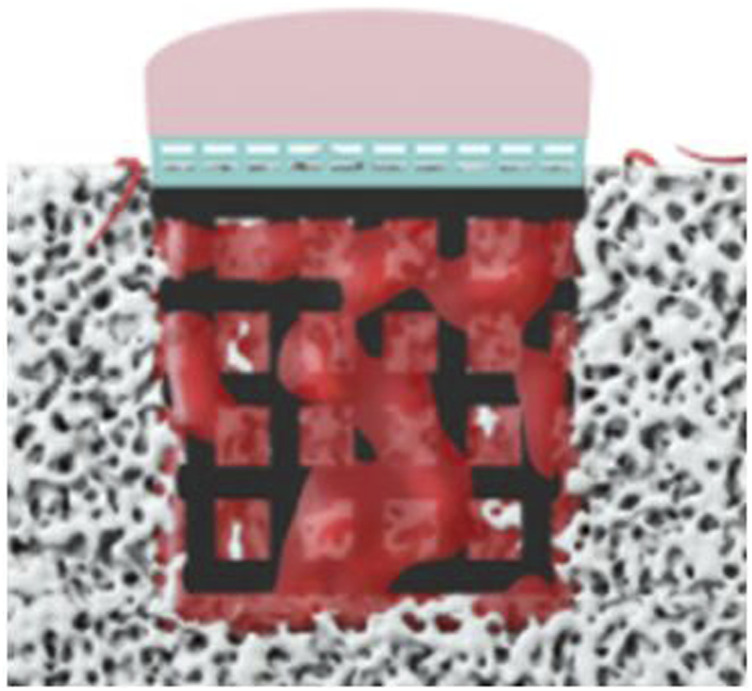
Boundary conditions of simulation (the bone marrow injected from the surrounding and bottom faces).

As the scaffold was pushed into the defect after the osteochondral defect was continuously washed, the simulation process is set as the bone marrow starts to flow into the defect when the scaffold is already stably put into the defect (Figure 5). In this model, as the scaffold roughness height is in the micro scale, the roughness height is set as 0. Nonslip and non-adherence conditions occurred on the wall and scaffold. Furthermore, as the scaffold manufacturing process is the same as our previous work, the surface and material settings are the same as the previous simulation model (Liu et al., 2020). To validate the cell distribution simulation by the *in vivo* test (osteogenesis on the scaffold), in this simulation, we assumed that BMSCs prefer to proliferate and differentiate on the scaffold surface.

This process is simplified to simulate the attaching process in which the bone marrow was set as coming from the surrounding and bottom faces for 2.5 s for filling the empty space. After that, two simulation working conditions are set to discover which boundary condition is more suitable for predicting cell attachment. One is that the scaffold starts to absorb cells after 2.5 s, and no more cells would be injected from the sides. The other one is to set at 1 mm/s injection speed of cells after 2.5 s.

The bone marrow is set as a non-Newtonian fluid governed by non-Newtonian power law. Consistency and power law index are set at k = 0.017 and n = 0.708, respectively, with 0.01 and 0.001 as their maximum and minimum viscosity limits. The bone marrow flowing speed is set at 1 mm/s, and the density is 1050 kg/m3. 50,000 spherical cells of 25 μm diameter are injected to the system for 2.5 s uniformly with 1,000 kg/m3 density and 0.03 N/s surface tension. Furthermore, cells could be attached on the boundary faces during the whole calculation period.

## Results

### Bionic Structural and Mechanical Performance of the Osteochondral Scaffold

As normal joints are formed by three parts, namely, the cartilage, subchondral bone plate, and trabecular bone, scaffolds were designed to mimic three layers of the bone in compliance with structural and mechanical properties. Considering only osteogenesis of the scaffold, the layer made by PLGA for cartilage regeneration would not be well-discussed in this article.

The bionic scaffold is designed with the PLA dense layer on the top and the titanium layer at the bottom which aims to mimic the osteochondral bone structure and mechanical property. The PLA dense layer plays the same role like the subchondral bone which could not only separate the synovial fluid and bone marrow but also connect the trabecular bone (the titanium layer of the scaffold). With high-porosity and interconnected holes, the titanium layer provides a structure for the tissue to attach and proliferate. Moreover, this 3D-printed titanium scaffold is characterized by a flexible design to match the requirement of the pore size, porosity, and surface area, which is used for supporting the mechanical loading from the sheep and provide a spatial structure for bone growth. The pore sizes larger than 100 μm are good for osteogenesis (Karageorgiou and Kaplan, 2005), and 100–400 μm are optimal for bone tissue regeneration (Hulbert et al., 1970; Schliephake et al., 1991; Bloebaum et al., 1994; Hofmann et al., 1997). According to a 3D printing machine’s accuracy and the residual stress after the printing, the beams of the scaffold were set at 0.5 mm in diameter, and pore sizes were set at 1 mm^2^.

The characterizations of the scaffold and bone are listed as follows (Table 1): To combine the PLA layer and titanium layer together, heat treatment was used to make these two layers stick together stably. As BMSC regeneration is hard to control *in vivo* or even the clinical trial and subchondral bone plate is hard to form as prospect, we believe that a dense layer of PLA could substitute biological and mechanical functions of the subchondral bone plate.

**TABLE 1:** Characterization of the scaffold and bone.

	*Pore size (mm)*	*Porosity*	*Surface area* (*mm* ^*2*^)	*Young’s modulus (MPa)*
***AX-10***	**—**	**—**	**157.95**	**1–2**
***Cartilage***	**—**	**—**	**—**	**0.3–1.5**Mow et al. (1984), Jurvelin et al. (1988), Arokoski et al. (1999), and Nieminen et al. (2000)
***PLA dense***	**—**	**—**	**112.68**	**2,200**
***Subchondral bone plate***	**—**	**—**	**—**	**635 ± 94** Wu et al. (2008)
***Titanium***	1*1 mm	**78.6%**	**411.67**	**70–100**
***Trabecular bone***	**1 mm**Kaplan et al. (1994), Keaveny et al. (2001), and Marks and Odgren (2002)	**50–90%**Kaplan et al. (1994), Keaveny et al. (2001), and Marks and Odgren (2002)	**—**	**19 ± 7** Mittra et al. (2005)

The layer for osteogenesis could not achieve the trabecular bone tissue structure as this kind of structure lacks mechanical properties and is easy to break. This layer is made of 3D-printed titanium layer cross-section. As some researchers mentioned that the synovial fluid inhibits bone formation (Andrish and Holmes, 1979; Hazelton et al., 1990), the biofunctional design that uses a PLA dense layer to avoid the synovial fluid flow into the trabecular bone section provides a reliable design for tissue regeneration.

### *In Vivo* Test

After being implanted into the sheep condyle, the bioperformance of the bionic osteochondral scaffold has been evaluated by consequent osteogenesis and tissue quality.

#### Quantification of Bone–Scaffold Contact

**FIGURE 6:**
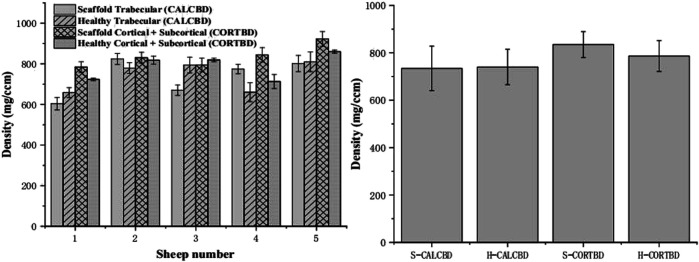
PQCT analysis of the trabecular and cortical BMD of the scaffold and healthy sheep condyle (**left:** each sheep; **right:** in total).

**FIGURE 7:**
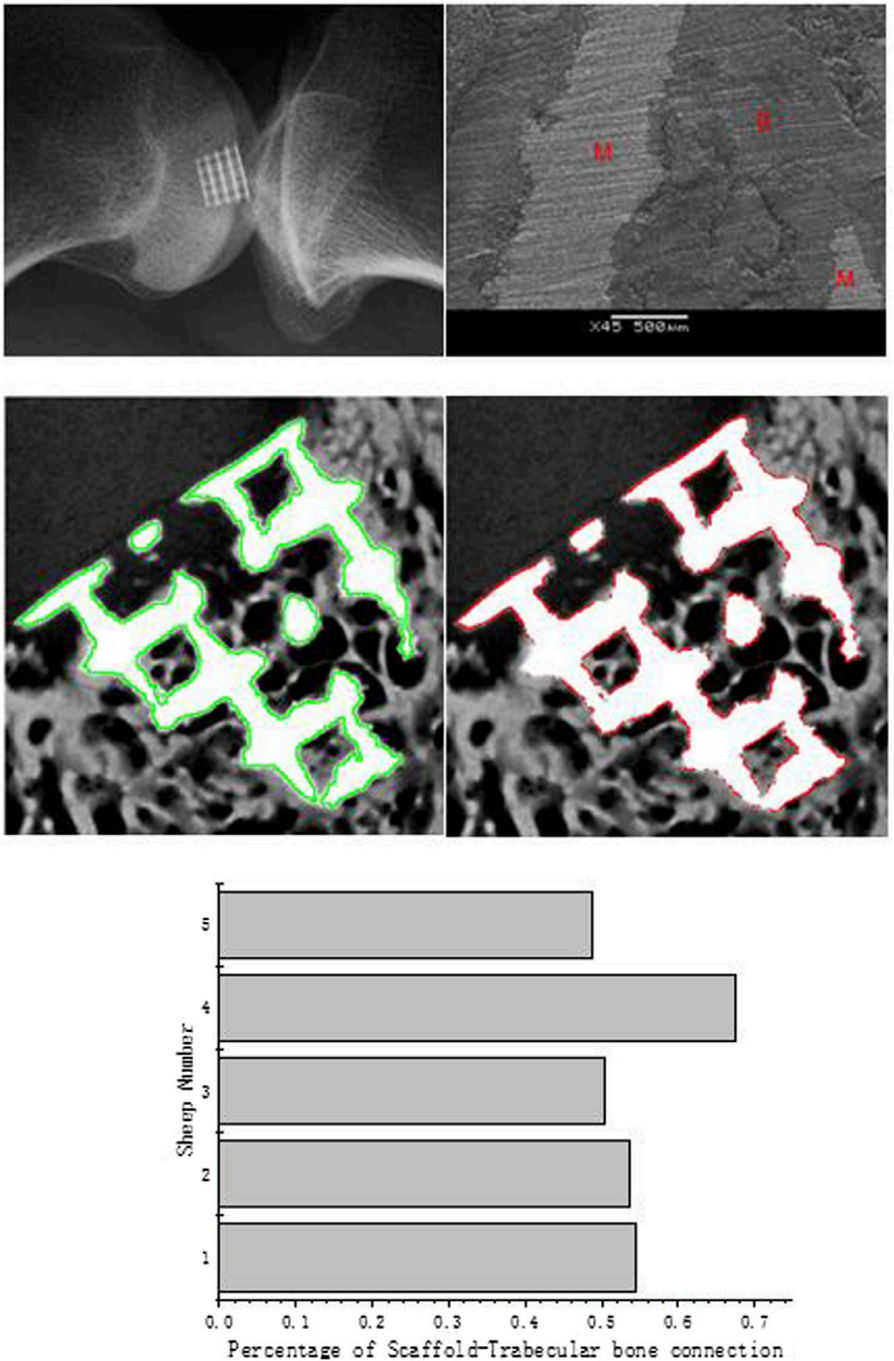
X-ray micrographs of the scaffold in the sheep condyle **(top left)**; an SEM image of scaffold–bone tissue connection **(top right;** M represents the metal alloy scaffold; B represents regenerated bone); the scaffold edge is identified by the green line **(medium left)**; bone–scaffold connection is identified by the red line **(medium right)**; percentage of scaffold–bone tissue connection **(bottom)**.

During the *in vivo* test, the 3D scaffold will be affected as the fixation becomes weakening by loading. Such weakening would sometimes occur during the implantation procedure (Knecht et al., 2007). After the surgery, four sheep gaits were normal, and only one sheep had a slight limping, but it recovered after several days. After a 3-month recovery period, all five sheep recovered well, and no postoperative complications were found during this period. The X-ray micrographs of the sheep condyle (Figure 6. left) showed that scaffold’s surrounding tissue connected well with the scaffold, and no loosening was seen in the image. According to the scanning electron microscope (SEM) image, it clearly illustrated that regenerated bone growth based on the metal surface connected well. To quantify and qualify the mechanical fixation, one of the most important things is to investigate tissue–scaffold interactions. Higher tissue–scaffold connection could provide higher skeletal integrity. It is shown that all the sheep's tissue–scaffold connections reached 50% and more after the 3-month healing process using the scaffold. One of them even nearly reached 70%. Because of the high-resolution X-ray image, some printing defects on the scaffold edge (powder with air) were identified, but it is hard to define whether it is the bone or scaffold. To make data more convincible, the real value of the connection of the bone and scaffold should be larger than it is shown in Figure 7.

According to the image analysis, we found that new bone tissues prefer to regenerate on the scaffold surface. Similar to the simulation boundary condition, the assumption has been proved by this analysis as the cells and tissues grew based on the scaffold surface.

#### Quantification of Osteogenesis

**FIGURE 8:**
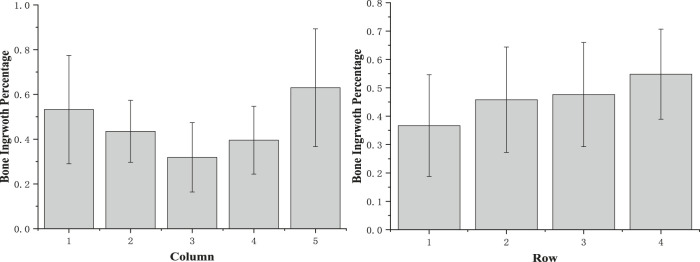
Osteogenesis of scaffold vertical and horizontal analysis.

To analyze the bone growth preference position in the scaffold, the grids were combined together as columns (horizontal) or rows (vertical). According to the results from the micro-CT and X-ray image analysis (Figure 8), in horizontal, compared with column groups 2 and 4, the side edge of the scaffold column group has more bone ingrowths (p1-2 = 0.457; p5-4 = 0.122). Also, the sub-mid area of column groups 2 and 4 is significantly larger than the middle area, with *p* values 0.247 and 0.452, respectively. No significant difference was found between columns 1–5 and 2–4. In the middle, more than 30% of the void space was occupied by the new regenerative bone tissue; around 40% on the subside of the scaffold and more than 50% of regenerative bone tissue occupied the space. Although the trabecular bone was regenerated more on the side of the scaffold than in the middle, it is believed that the decreasing trend from the side to middle is very slight. Vertically, a slightly decreasing trend was observed from the bottom to top surface. Nearly no difference of the regenerative bone formation was observed between two middle rows. Although the regenerated bone tissue showed a gradual growth trend from the scaffold edge to the middle, the growth rate in the scaffold middle is considerable.

#### Bone Mineral Density

At 3 months, bone mineral density (BMD) of the cortical bone for the scaffold group and healthy group was 835.04 ± 87.46 and 784.98 ± 72.32 mg/cm^3^, respectively. The trabecular bone density of the scaffold and healthy groups was 734.44 ± 131.01 and 740.18 ± 81.98 mg/cm^3^, respectively, as shown in Figure 6. There were no significant interactions between scaffold and healthy groups’ BMD on either the trabecular bone or cortical bone density. Mean trabecular BMD was not significantly different between the healthy knee and experimental knee (*p* = 0.92). And also, no significant difference was found in mean cortical and subcortical BMD (*p* = 0.24).

### Simulation of Bone Mesenchymal Stem Cell Attachment on the Scaffold

**FIGURE 9:**
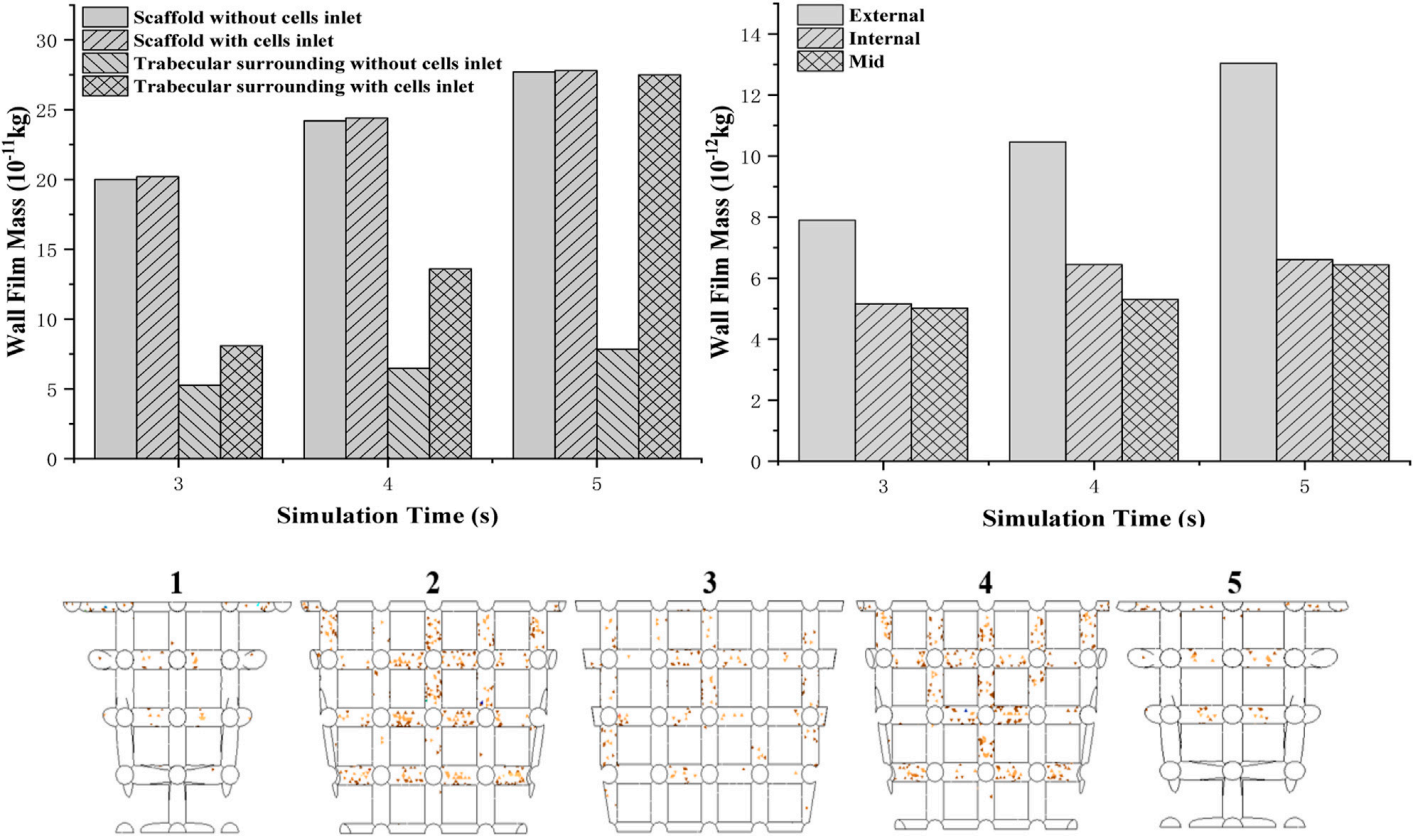
Two different boundary conditions of cells attached to a mass of scaffold and boundary wall **(top left)**; cell (density) distribution in the scaffold from the edge to the middle **(top right)**; simulation of cell attachment at 3 s—boundary conditions: no cells injection **(bottom)**.

This simulation aims to investigate the cell attachment process and the final cell distribution after scaffolds are put into the defect, and the bone marrow flows into the defect void space through combining DPM and VOF model. According to the boundary conditions, cells are not attached to the scaffold at the first 2.5 s to mimic the real circumstance of the *in vivo* test. Then, the cell attachment mass counts every integer time from 3 to 5 s. For the whole osteochondral defect system, cell density of scaffolds in two boundary conditions with or without cell inlet after 2.5 s showed a steady increase (Figure 9 top left). However, as for boundary faces’ (trabecular bone) cell density, it showed a rapid growth when cells were still coming to the system after 2.5 s. At 5 s, the cell density of surroundings even reached the same value as the scaffold.

To validate the *in vivo* test more accurately, the scaffold is separated to five parts from one side to the other. Cell attached mass is accounted by averaging the mass on each beam. It is found that from the external to the middle of the scaffold, the cell attached mass showed a decreasing trend (Figure 9 top right). Compared to the external’s cell density which increased rapidly when time goes by, only a little increase in cell density for the internal and middle areas is shown in the graph.

Separating into five parts to investigate cell distribution thoroughly (Figure 9 bottom), it is found that fewer cells attached at the bottom beam even though they were closer to the bottom injection face. Moreover, sub-closer to edge parts 2 and 4 have more cells attached than 1 and 5.

## Discussion

In recent years, cell-based repairing therapeutics has been proved not successful enough for patients who have OA or cartilage defects (Fellows et al., 2016). Lack of blood vessels, low chondrocyte density, and migration ability make cartilage hard to repair (Henrotin et al., 2005). Compared to the autologous chondrocyte implantation (ACI) technology, using scaffolds to recruit BM-MSCs is seen as an effective way for cartilage regeneration and repair.

An ideal three-dimensional scaffold needs to have the same geometrical, mechanical, and biological properties with the host tissue. Our novel bionic osteochondral scaffold is designed by mimicking three main parts of the osteochondral bone which are cartilage, subchondral bone plate, and trabecular bone (Burr and Gallant, 2012). To mimic cartilage’s mechanical and biofunctional properties, we combined PLGA and collagen together with a 1–2 MPa elastic modulus. Although the peak stresses *in vivo* could be reached 18 MPa in joints during dynamic loads (Hodge et al., 1986), the elastic modulus is typically 0.3–1.5 MPa (Mow et al., 1984; Jurvelin et al., 1988; Arokoski et al., 1999; Nieminen et al., 2000).This phenomenon is caused by highly pressurized interstitial water during dynamic loading as it cannot be squeezed out during the loading process, which could cause high physiological stresses (Jurvelin et al., 1997; Laasanen et al., 2003). The high-porosity inner structure could also achieve the same function. As for the subchondral plate, it plays an important role in supporting the cartilage and also separating the synovial fluid and trabecular bone because osteogenesis could be inhibited by the synovial fluid (Andrish and Holmes, 1979; Hazelton et al., 1990). The PLA layer design is able to prevent the synovial fluid flow into the trabecular bone defect during the surgery and healing process and also provide sufficient mechanical support. The bottom layer mimicking the trabecular bone is made by 3D-printed titanium which is widely used for trabecular bone regeneration because of its excellent biocompability, mechanical property, chemical stability, and suitability to mimic the biomimetic geometry (Long and Rack, 1998). The trabecular bone, with lower resistance to stress (50 MPa) and high resistance to strain (50%), has 50–90% porosity (Kaplan et al., 1994) and 1 mm diameter pore size (Keaveny et al., 2001). As 100–400 μm pore size is seen as optimal for bone regeneration (Hulbert et al., 1970; Schliephake et al., 1991; Bloebaum et al., 1994; Hofmann et al., 1997), a scaffold with 78.6% porosity and nearly 500 μm is designed.

As the porous design could result in diminished mechanical properties, the design needs to have an adequate mechanical stability to enable initial fixation with the host tissue during implantation as well as surface loading after surgery (Knecht et al., 2007). If the implantation detached partially or even completely fails *in vivo*, patients would feel serious locking or catching at the target area (Nehrer et al., 1999; Peterson et al., 2000). According to the *in vivo* test analysis by X-ray and SEM, it is found that new regenerated tissue connected to the scaffold with high porosity with similar mechanical property to natural bone tissue is really stable.

Interestingly, we found that even the regenerated bone tissue does not fill the void space of the bone defect after 3 months; more than 50% of the scaffolds’ whole structure is surrounded by new bone tissue, and there is only a slight decrease in osteogenesis from the side to the middle. This phenomenon illustrates that tissues and cells grow based on the scaffold surface which also points out that cell and tissue distribution is really important as good osteogenesis provides high-quality cartilage.

**FIGURE 10:**
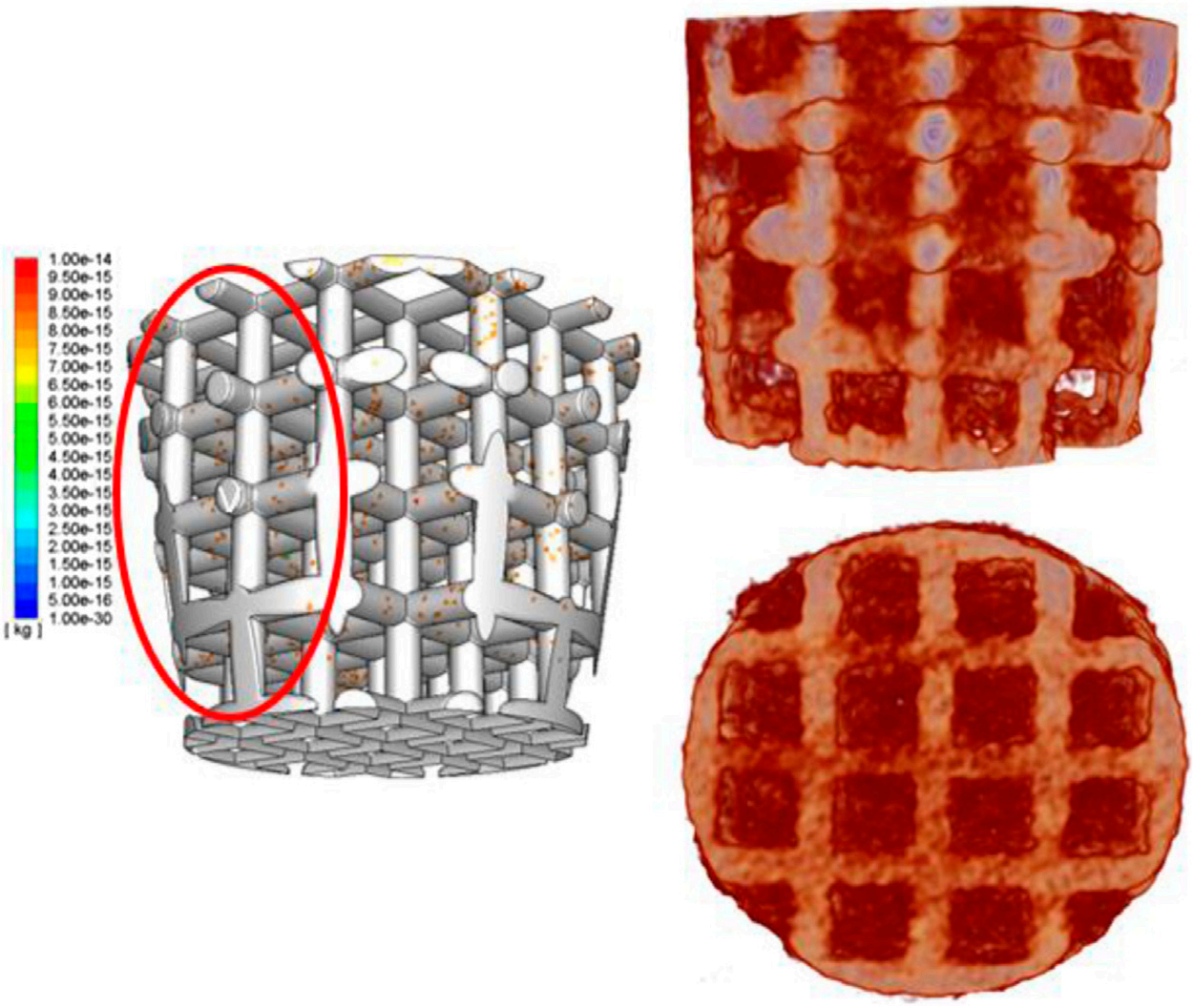
Simulation results of cell distribution in a 3D structure **(left)**; 3D-reconstructed image of the scaffold and bone tissue ingrowth **(right)**.

To further investigate cell distribution in the scaffold for *in vivo* tests, as it is impossible to sacrifice animals at the initial stage (Keaveny et al., 2001), a novel numerical model has been developed to predict cell distribution after the surgery process. For validating the model, we compared the cell distribution results from simulation in 5 s and the regenerated bone distribution of the sheep after 3 months together. According to previous research of Byrne et al., cell proliferation, differentiation, and migration just occur in neighboring areas (Keaveny et al., 2001), and it is found that cell migration does not influence a lot in cell distribution, especially in macroscale (Keaveny et al., 2001). In that case, it is believed that cell distribution at the initial stage is really related to the final bioperformance—regenerated tissue distribution (Keaveny et al., 2001). In other words, it is found that cells remain in their position on the scaffold when they work together and form tissues, as shown in Figure 10. As for the bone tissue distribution, horizontally, the trabecular bone occupied more percentage of the void volume at an external place than in the middle, which showed a same trend as external beams have more cell density after cell attachment. But the simulation results did not show that all external areas have really high cell density as *in vivo* tests showed. The reason is that cells attaching to the previous defect hole surfaces cannot be avoided. The cells attaching on the surroundings would proliferate and differentiate to build up the tissues. That is why the external place showed a more intensive cell density. In addition, comparing the cell distribution vertically between the experiment and simulation did not show that the bottom area has higher trabecular bone growth than the top. However, the cell density of the simulation only shows the same trend of the experiment results after the second titanium junction layer. It is clearly seen that not many cells were attached to the lower beam during the attaching process. But, a lot of cells were attached to the bottom surface of the hole. The bone percentage of the bottom is larger than that of the top because the bottom surface has much cell attachment, and they can absorb nutrients more easily as their position is much more close to the bottom and surrounding surfaces (nutrients injection position). In that case, the boundary condition that cells still come to the system after the bone marrow fills the void volume is more accurate to simulate the initial stage of tissue engineering—recruiting cells.

After validation and finding the best boundary condition to simulate the cell attachment process, we found that during the whole attachment process, the external area’s cell density was increasing rapidly, but the cell density of internal and middle areas increased extremely slowly. The reason is that initially (first 2.5 s), cells distribute more uniformly as the bone marrow already fills the void space and become steady at that time. When the scaffold starts to absorb cells (after 2.5 s), at the beginning, the cells attaches to the scaffold uniformly. After that, the balance of the cell density in the fluid breaks down, and cells start to move. As there are many beams in the middle providing drags for cells to move in, cells prefer to move outside to somewhere with less drags. That is why the cell density at external beams increases quicker than in the middle.

This novel numerical method for predicting tissue distribution is also available to provide rehabilitation guidance for patients after the surgery as the inner environment of the knee joint like flow speed and temperature could be influenced by rehabilitation devices. By this model, the optimized flow speed and temperature could be explored, and some special medical device should be designed to achieve this environment.

There are some drawbacks to the experiments. First, it is impossible to recognize whether the surrounding areas’ bone tissue is a new regenerate bone tissue or old ones according to the X-ray images. Although the external bone tissue growth is well, we cannot identify whether its growth is mainly based on the scaffold influence or it is just the surrounding bones’ remodeling and regeneration.

Although we could only ensure that the bone tissue in the internal scaffold’s pores are the new tissues, the biomimic scaffold still showed good-quality bioperformance. Second, during the drilling osteochondral defect process, it is unavoidable that the old bone’s powder created by the driller in the surroundings may be pushed into the bottom when the scaffold is pushed into the hole. In that case, the bone percentage in the bottom is higher than the other areas, which may not only be influenced by the cell attachment. Bone resorption and remodeling would also affect the final results.

## Conclusion

This study develops a numerical model which could help researchers to optimize scaffold material property and geometry and could also avoid unnecessary *in vivo* and *in vitro* tests. As the initial healing process of the sheep could not be observed accurately, this model provides an opportunity for researchers to understand how the cell attached and distributed influenced by the scaffold structure and a proper mechanical and biological environment. The simulation results of cell distribution in the scaffold matched well with the regenerated bone tissue distribution in the *in vivo* test. The regenerated bone tissue on the surroundings (scaffold–material interface) is only around 15% more than that in the inner structure. This model could achieve an application to design a personalized scaffold and provide a proper mechanical and biological environment for recovery. It is also useful to help surgeons provide rehabilitation guidance for patients after implantation considering their knee joint inner environment.

## Data Availability

The original contributions presented in the study are included in the article/Supplementary Material. Further inquiries can be directed to the corresponding authors.
